# Systemic mapping of organ plasma extravasation at multiple stages of chronic heart failure

**DOI:** 10.3389/fphys.2023.1288907

**Published:** 2023-11-16

**Authors:** Oliver Kitzerow, Paul Suder, Mohanad Shukry, Steven J. Lisco, Irving H. Zucker, Han-Jun Wang

**Affiliations:** ^1^ Department of Genetics Cell Biology and Anatomy, University of Nebraska Medical Center, Omaha, NE, United States; ^2^ Department of Anesthesiology, University of Nebraska Medical Center, Omaha, NE, United States; ^3^ Department of Cellular and Integrative Physiology, University of Nebraska Medical Center, Omaha, NE, United States

**Keywords:** inflammation, chronic heart failure, pancreas, gastro-intestinal dysfunction, pulmonary hypertension

## Abstract

**Introduction:** Chronic Heart failure (CHF) is a highly prevalent disease that leads to significant morbidity and mortality. Diffuse vasculopathy is a commonmorbidity associated with CHF. Increased vascular permeability leading to plasma extravasation (PEx) occurs in surrounding tissues following endothelial dysfunction. Such micro- and macrovascular complications develop over time and lead to edema, inflammation, and multi-organ dysfunction in CHF. However, a systemic examination of PEx in vital organs among different time windows of CHF has never been performed. In the present study, we investigated time-dependent PEx in several major visceral organs including heart, lung, liver, spleen, kidney, duodenum, ileum, cecum, and pancreas between sham-operated and CHF rats induced by myocardial infarction (MI).

**Methods:** Plasma extravasation was determined by colorimetric evaluation of Evans Blue (EB) concentrations at 3 days, ∼10 weeks and 4 months following MI.

**Results:** Data show that cardiac PEx was initially high at day 3 post MI and then gradually decreased but remained at a moderately high level at ∼10 weeks and 4 months post MI. Lung PEx began at day 3 and remained significantly elevated at both ∼10 weeks and 4 months post MI. Spleen PExwas significantly increased at ∼10 weeks and 4 months but not on day 3 post MI. Liver PEx occurred early at day 3 and remain significantly increased at ∼10 weeks and 4 months post MI. For the gastrointestinal (GI) organs including duodenum, ileum and cecum, there was a general trend that PEx level gradually increased following MI and reached statistical significance at either 10 weeks or 4 months post MI. Similar to GI PEx, renal PEx was significantly elevated at 4 months post MI.

**Discussion:** In summary, we found that MI generally incites a timedependent PEx of multiple visceral organs. However, the PEx time window for individual organs in response to the MI challenge was different, suggesting that different mechanisms are involved in the pathogenesis of PEx in these vital organs during the development of CHF.

## Introduction

Chronic heart failure (CHF) is a highly prevalent disease that drastically increases morbidity and mortality of patients (Roger, 2021). It is estimated that CHF impacts over 37 million people worldwide and is the leading cause of hospitalization among adults and the elderly (Ziaeian and Fonarow, 2016). CHF occurs secondary to earlier cardiac and vascular disorders such as hypertension, myocardial infarction (MI), or myocarditis (Kemp and Conte, 2012). Post-MI timelines of cardiac inflammation and fibrosis have been extensively analyzed as have their effects on cardiac morphology and function (Cleutjens et al., 1995; Sutton and Sharpe, 2000; Frangogiannis, 2012; Prabhu et al., 2016; Talman and Ruskoaho, 2016). What remains unknown are the influences of CHF on extracardiac organs at different timepoints during the disease. Assessing extracardiac organ function will provide a more comprehensive understanding of pathogenesis and comorbidities of CHF and promote the development of novel therapies. In this study, we performed permanent coronary ligation MI to investigate the impact of early, middle, and late stages of CHF on thoracic and abdominal organ plasma extravasation (PEx) in rats.

All cells require nutrients and a means of removing waste. In higher organisms, these functions are primarily carried out by the circulatory system. Although the vascular system is considered closed, it must be adequately permeable to allow for the exchange of nutrients and waste products. Under basal vascular permeability (BVP), molecules are exchanged in the capillaries and consist largely of water, gases, glucose, and slight amounts of plasma proteins (McDonald et al., 1999; Nagy et al., 2008). Permeability can be described in 2 other pathological forms: acute vascular hyperpermeability (AVH) and chronic vascular hyperpermeability (CVH), both of which contribute to the pathogenesis of PEx. In the instances of AVH or CVH, extravasated fluid, known as exudate, is protein rich, approaching concentrations levels of plasma. Notably, AVH is self-limiting, but chronic exposure to factors that increase permeability results in profound changes in venular structure such as pericytes detachment and endothelial thinning. The result is an enlarged highly permeable venule with profound exudate. The subsequent protein accumulation and osmotic pressure in the interstitium drives fluid retention and promotes edema. In addition, plasma proteins can interact with tissue factors to induce migration and activation of mesenchymal and inflammatory cells (Colvin and Dvorak, 1975; Dvorak et al., 1979). These actions can limit the diffusion of nutrients and elimination of waste and ultimately impair organ function. Therefore, determining PEx of a given tissue or organ can potentially serve as a guide for organ health. However, the time course of PEx in individual organs at the different stages of CHF has not been previously established. In this study, we utilized EB to assess the degree of PEx. EB binds and stains albumin, so tissues undergoing PEx are stained bluish purple due to the entrapped EB-albumin complex within their parenchyma. Concentrations of EB can then be calculated to determine relative levels of PEx and predict the course of hyperpermeability. The goals of this study were to quantify the levels of PEx in various organs at multiple timepoints after the induction of MI in hopes to determine the effects of multiple stages of CHF on extracardiac organ permeability.

## Methods

All animal experimentation was approved by the Institutional Animal Care and Use Committee of the University of Nebraska Medical Center and performed in accordance with the National Institutes of Health’s Guide for Use and Care of Laboratory Animals and in accordance with the ARRIVE guidelines (Kilkenny et al., 2012; Institute of Laboratory Animal Resources, 2021). Experiments were performed on adult, male Sprague-Dawley rats purchased from the Charles River Laboratories (Wilmington, MA). Food and water were supplied *ad libitum*, and rats were kept on 12-h light/dark cycles.

### Rat model of chronic heart failure

Rats (180–200 g) were chosen at random to undergo either MI surgery or sham surgery. CHF was produced by left coronary artery ligation as previously described (Hong et al., 2021). Sham-operated rats were prepared in the same manner but did not undergo coronary artery ligation. Briefly, the rat was ventilated at a rate of 60 breaths/min with 2%–3% isoflurane during the surgical procedure. A left thoracotomy was performed through the fifth intercostal space, the pericardium was opened, the heart was exteriorized, and the left anterior descending coronary artery was ligated. For post procedure pain management, Buprenorphine SR (1.5 mg/kg) was subcutaneously injected immediately after surgery. All sham rats survived, and∼70% of rats survived from coronary artery ligation surgery. At the end of terminal experiments, infarct size (IS) was measured by taking the ratio of the infarct area to whole left ventricle (LV) minus septum in rats 10 weeks or 4 months post sham/MI. In rats 3 days post sham/MI, we did not measure IS because the scar has not formed although each infarcted heart exhibited a pale ischemic region.

### Tissue plasma extravasation and quantification of Evans Blue

Rats were anesthetized with urethane (800 mg/kg ip) and *α*-chloralose (40 mg/kg ip). EB, 20 mg/kg (10 mg/mL, dissolved in heparinized saline) was administered intravenously through the femoral vein. After 10 min, anesthetized rats were euthanized by transcardiac perfusion with Phosphate Buffered Saline (0.01 M, pH 7.4). Organ tissues including lungs, heart, kidney, liver, pancreas, duodenum, ileum, cecum, and spleen were excised, weighed, and photographed. Additional dissection of infarcted hearts was performed to separate regions of the infarct. The left ventricle was dissected into scar, peri-scar tissue that was adjacent the scar, and remote tissues. Then, samples were placed in 2 mL of N,N′-dimethyl formamide, homogenized, and incubated at 50°C water bath for 24 h. Samples were then centrifuged (1 min, 14,000 rpm) and the EB content in the supernatant was determined in a 96-well microplate reader (infinite M200, TECAN, Männedorf, CH, Switzerland) using a fluorescence excitation wavelength of 620 nm (bandwidth 10 nm) and an emission wavelength of 680 nm (bandwidth 40 nm) (100 µL sample per well). Extravasation of EB was expressed as mg EB/g of tissue, by comparing the experimental values with a known standard.

### Statistics

Statistical evaluation was analyzed using GraphPad Prism (GraphPad Software, San Diego, CA. Version 9.4.1). Normality was first determined using the Shapiro-Wilk test with an alpha set at 0.05. If organ data sets were not normally distributed, Mann-Whitney tests were utilized to determine differences between sham and MI groups. Unpaired t-tests were used when data sets were normally distributed. The comparison of PEx between each timepoint for MI rats was done using one-way ANOVA with tukey’s multiple comparison test with *p* < 0.05 being statistically significant. Differences between two groups (Sham and MI) were defined at *p* < 0.05 being statistically significant.

## Results

### Evaluation of cardiac PEx at different stages of CHF

**FIGURE 1:**
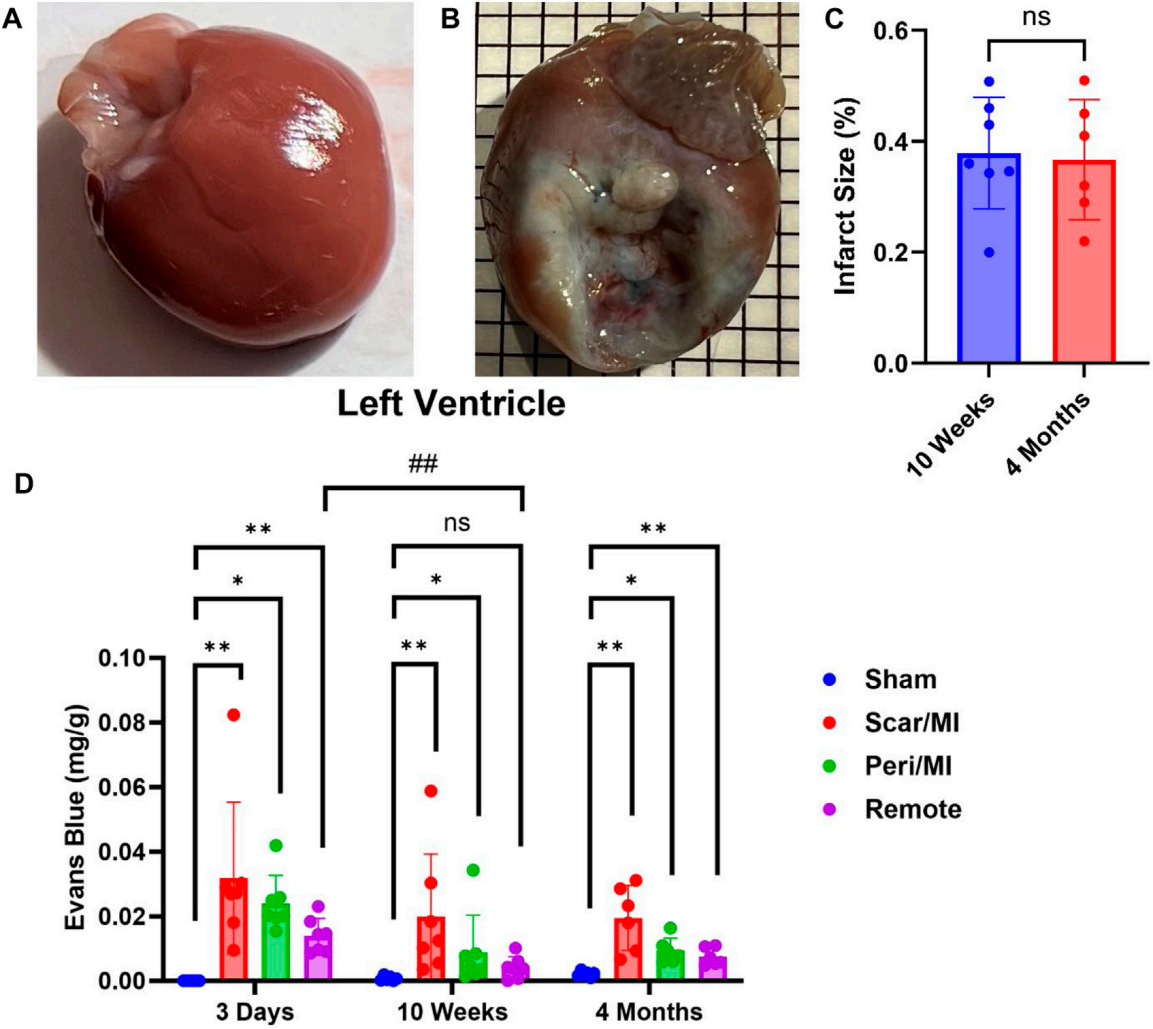
Time-dependent PEx in the heart post sham/MI. 10 weeks post MI shows scar formation and PEx visualized by the tissue content of EB in the MI **(B)** heart but not sham **(A)**. Extent of scar area is shown in **(C)**. Time course of PEx in various regions of the left ventricle in control and MI animals **(D)** Mean ± SD. n = 5-8 per group. Comparing MI with Sham group *p* < 0.05 (*) and *p* < 0.005 (**). Comparing timepoints within MI group *p* < 0.005 (##).

Infarcted rats at 10 weeks (i.e., middle stage of CHF) and 4 months (i.e., late stage of CHF) post MI showed significant scaring of the left ventricle compared to sham operated rats (IS at 10 weeks post MI: 37.8% ± 9.3%; IS at 4 months post MI: 36.7% ± 9.9%) (Figure 1C). All regions of the left ventricle experienced significant PEx with the scar tissue undergoing the most. At 10 weeks the remote region was significantly decreased compared to 3 days and did not show significant PEx when compared to sham. The Peri-MI region experiences PEx through 3 days and up to 4 months when compared to control. Surprisingly, the scar tissue showed the same pattern to a greater extent (Figure 1). These data demonstrate that PEx in cardiac tissues occurs at all stages of CHF, but the level at which it occurs depends on stage of CHF and proximity to the area of effect.

### Evaluation of lung PEx at different stages of CHF

**FIGURE 2:**
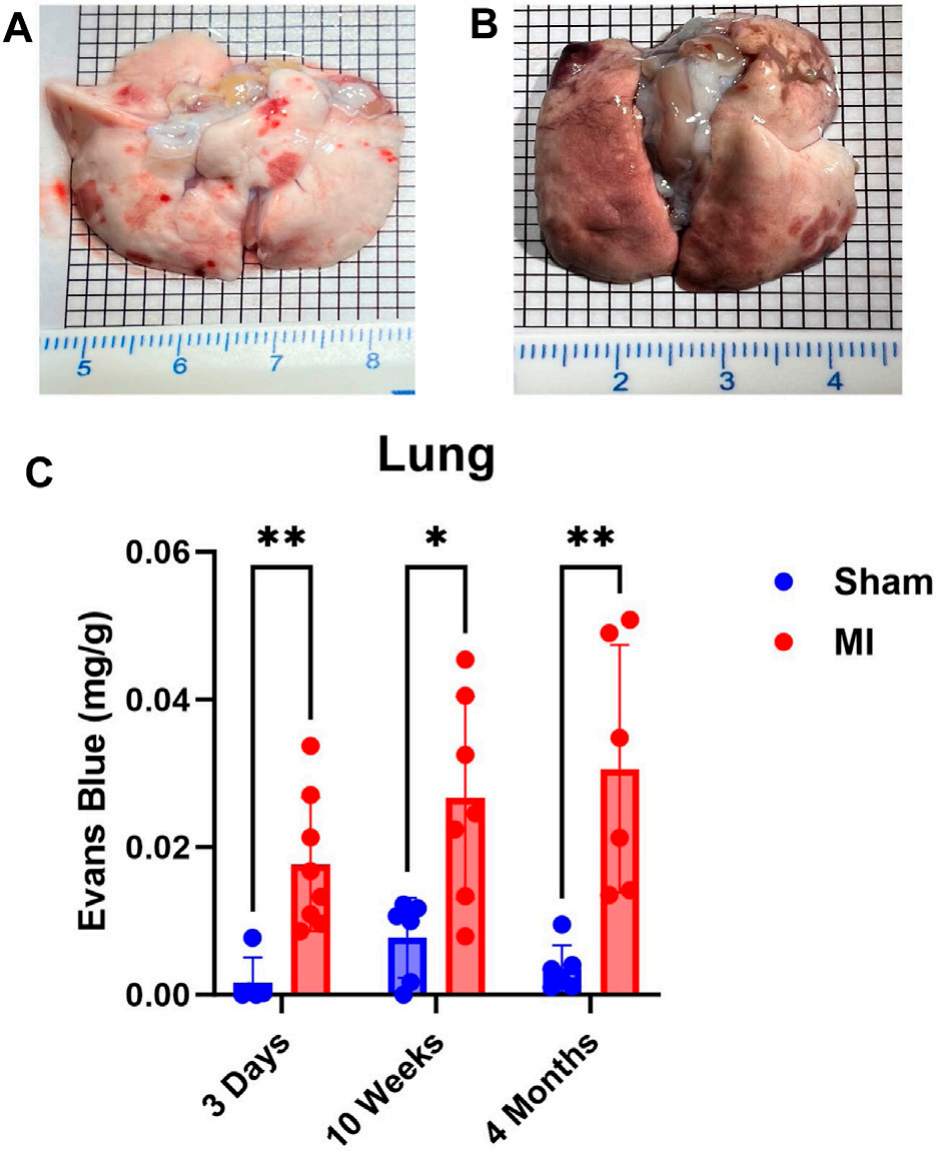
Time-dependent PEx in the lungs post sham/MI. Representative images of sham **(A)** and MI **(B)** lungs treated with EB at 10 weeks post-surgery. Time course of PEx in the lung post MI **(B)** and sham surgery **(C)**. Mean ± SD. n = 5-8 per group. Comparing MI with Sham group *p* < 0.05 (*) and *p* < 0.005 (**).

Next, we examined lung PEx at different stages of CHF. Due to the relative proximity of the lung to the heart and their interconnected functions, we hypothesized the lung would mirror the temporal PEx patterns as the heart. As expected, the lungs started showing significant levels of PEx at 3 days post MI. Significant levels of PEx were seen through 10 weeks and 4 months post MI similar to the heart (Figure 2). Contrary to the heart, PEx in the lungs did not decrease over time.

### Evaluation of gastrointestinal organ PEx at different stages of CHF

**FIGURE 3:**
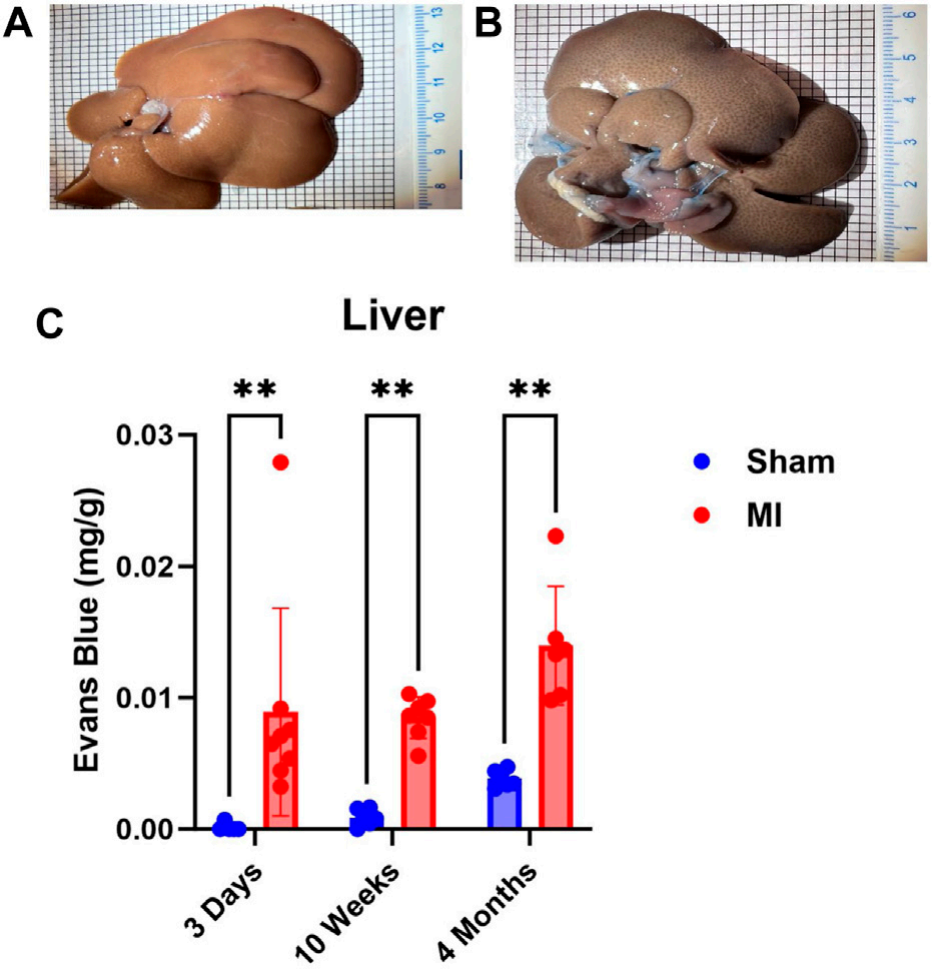
Time-dependent PEx in the liver post sham/MI. Representative images of sham **(A)** and MI **(B)** liver treated with EB at 10 weeks post-surgery. Time course of PEx in the liver PEx in sham and MI animals post-surgery **(C)**. Mean ± SD. n = 5-8 per group. Comparing MI with Sham group *p* < 0.005 (**).

**FIGURE 4:**
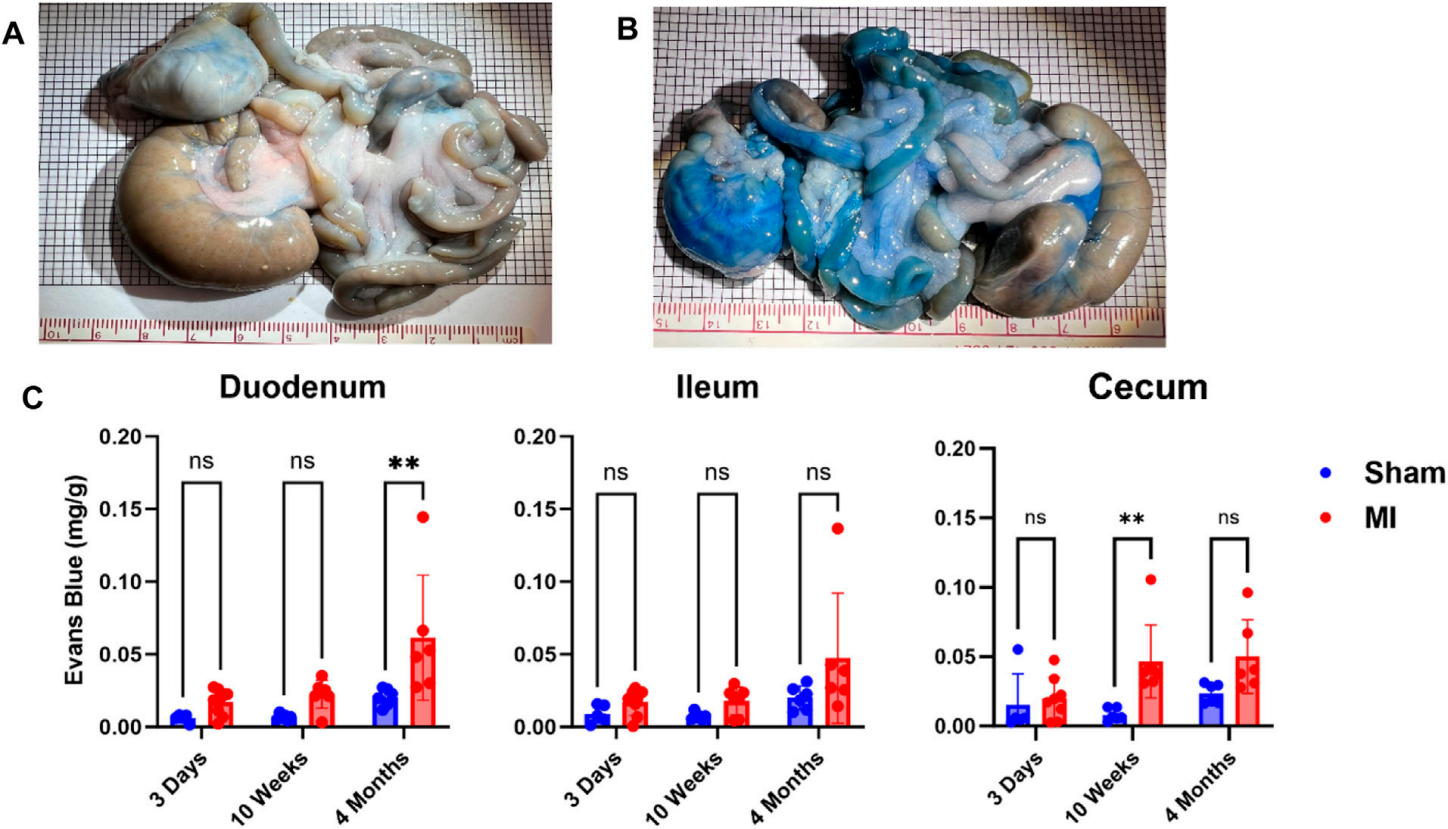
Time-dependent PEx in the gastrointestinal (GI) tract post sham/MI. Representative images of sham **(A)** and MI **(B)** GI tract treated with EB at 4 months post-surgery. Time course of PEx in the areas of the GI tract from sham and MI animals post-surgery **(C)**. Mean ± SD. n = 5-8 per group. Comparing MI with Sham group *p* < 0.005 (**).

**FIGURE 5:**
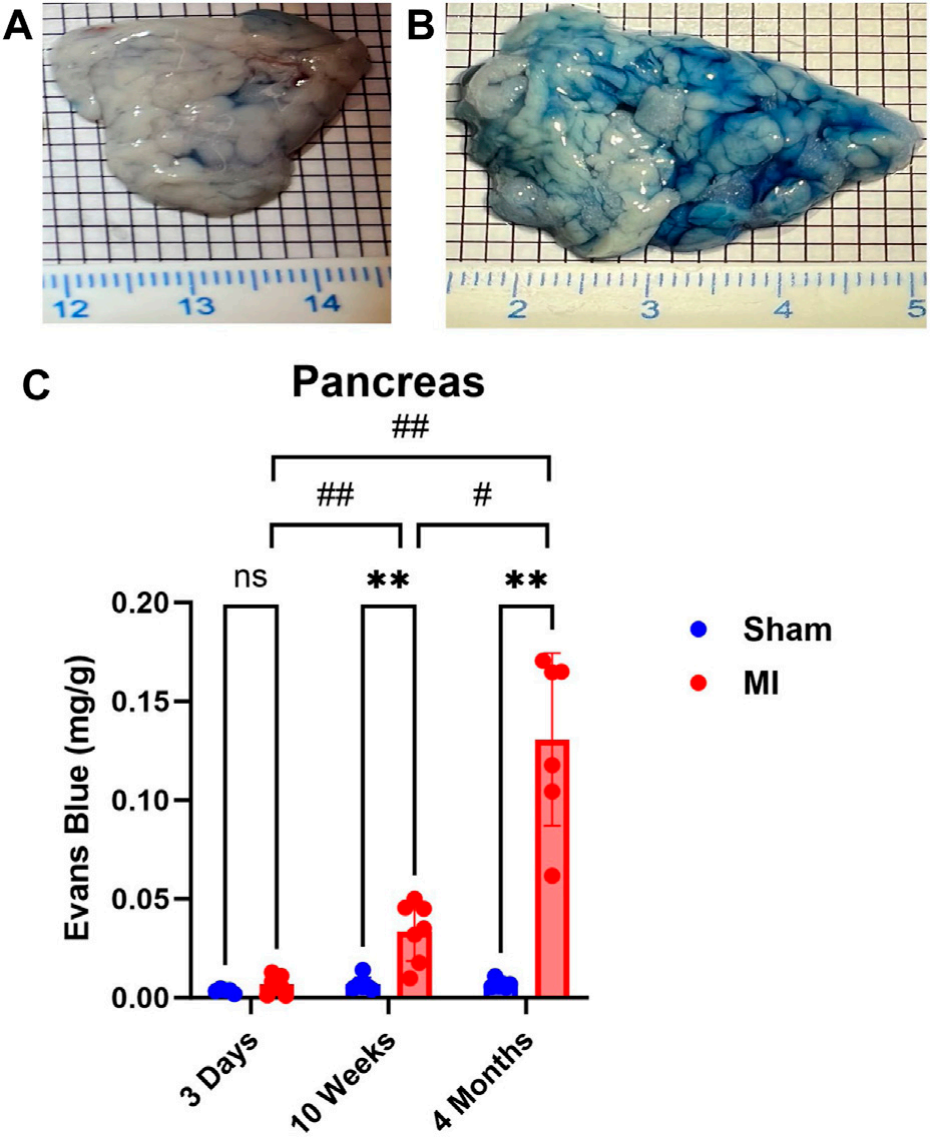
Time-dependent PEx in the pancreas post sham/MI. Representative images of sham **(A)** and MI **(B)** pancreas treated with EB at 10 weeks post-surgery. Time course of PEx in the areas of the GI tract from sham and MI animals post-surgery **(C)**. Mean ± SD. n = 5-8 per group. Comparing MI with Sham group *p* < 0.005 (**). Comparing timepoints within MI group *p* < 0.05 (#) and *p* < 0.005 (##).

Pulmonary hypertension and right-sided heart failure often precede liver dysfunction. We predicted that significant levels of liver PEx would follow lung PEx. However, significant liver PEx occurred at 3 days and continued to 4 months post MI (Figure 3). Due to the hepatic portal system, we predicted concurrent and subsequent PEx of the digestive tract. However, only the duodenum and cecum showed significant PEx, and notably at varying timepoints (Figure 4). The cecum experienced significant PEx at 10 weeks while the duodenum reached significant levels at 4 months. There appeared to be slight enhancement of PEx at 4 months post MI in ileum and cecum as well, but these were not statistically significant. The pancreas did not experience significant PEx until 10 weeks and continued through 4 months (Figure 5). In contrast to the other digestive organs, PEx of the pancreas increased significantly over time with levels tripling from 10 weeks to 4 months.

### Evaluation of spleen PEx at different stages of CHF

**FIGURE 6:**
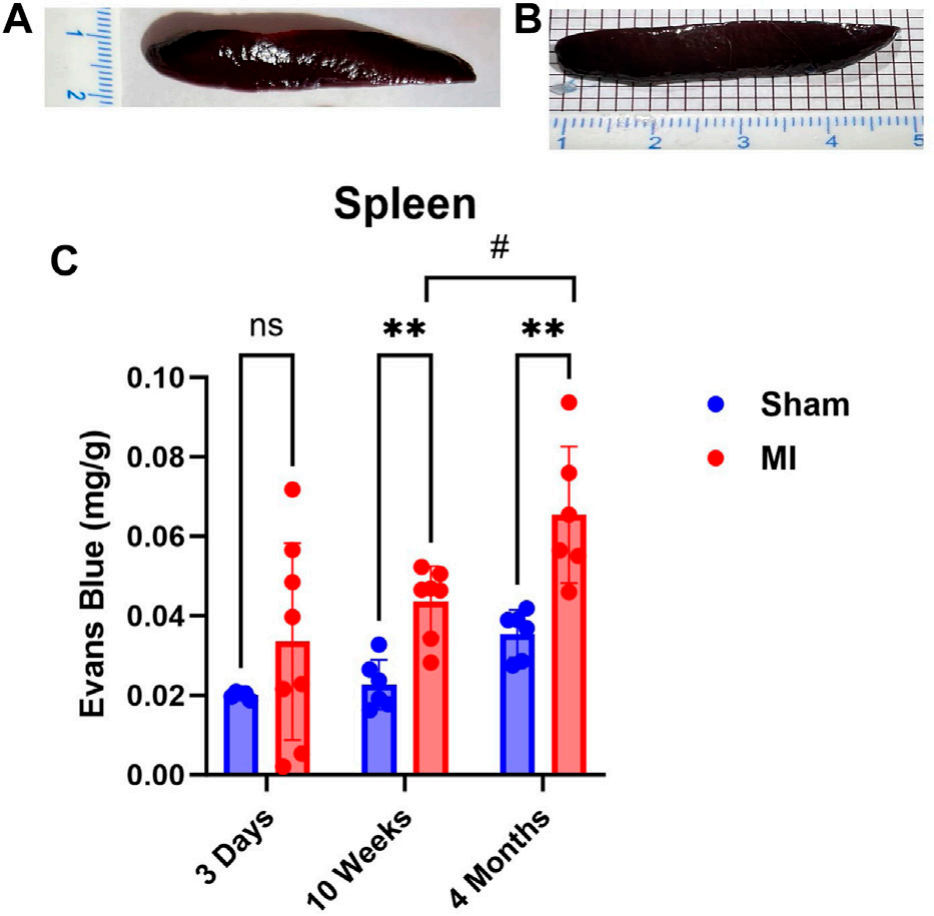
Time-dependent PEx in the spleen post sham/MI. Representative images show PEx in the spleen at 10 weeks post sham/MI **(A,B)**. Time course of PEx in the spleen from sham and MI animals post-surgery **(C)**. Mean ± SD. n = 5-8 per group. Comparing MI with Sham group *p* < 0.005 (**). Comparing timepoints within MI group *p* < 0.05 (#).

The spleen is a secondary lymphoid organ that houses leukocytes, which can be mobilized in the event of tissue damage or infection. As such, we expected significant levels of PEx to occur acutely and chronically. Surprisingly, PEx was not observed until week 10 post MI (Figure 6). Also, much like the pancreas, PEx of the spleen worsens at 4 months post MI.

### Evaluation of renal PEx at different stages of CHF

**FIGURE 7:**
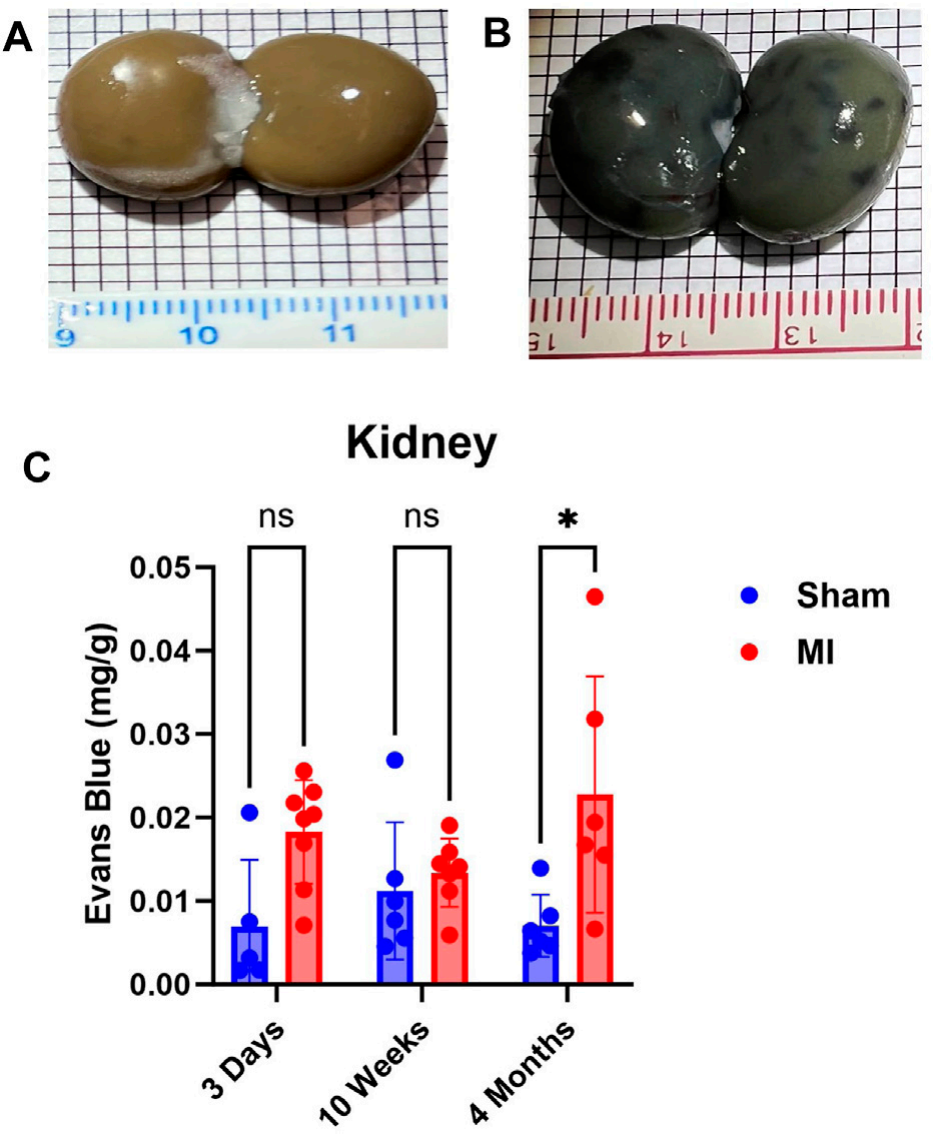
Time-dependent renal PEx post sham/MI. Representative images show PEx in the kidney at 4 months post sham/MI surgery **(A, B)**. Time course of PEx in the kidney from sham and MI animals post-surgery **(C)**. Mean ± SD. n = 5-8 per group. Comparing MI with Sham group *p* < 0.05 (*).

The kidney did not show significant levels of PEx at 3 days post MI (Figure 7). PEx levels were still normal at 10 weeks but increased at 4 months post MI. These data closely resemble previously documented compensatory actions of the kidney at the mid-stage of CHF (Xia et al., 2022). The late renal PEx is consistent with declined renal function at the late stage of CHF.

## Discussion

In this study, we examined multiorgan PEx at different stages of CHF. We used an MI model for the development of CHF in rats. MI can produce left ventricular remodeling including changes in LV size, shape, function, and cellular and molecular composition (Sutton and Sharpe, 2000). By utilizing EB, we determined divergences of PEx in thoracic and abdominal organs, the severity, and initiation and duration. In general, we found that MI induces a time-dependent increase of PEx in most visceral organs in addition to the heart. However, due to varying timepoints of PEx initiation and duration the mechanisms that promote such extravasation most likely differ among these organs.

The tight regulation of protein extravasation helps to maintain the oncotic pressure of both the blood and tissue interstitium, thus sustaining homeostasis. In general, vascular permeability (VP) is the ability of fluid to move across microvessels to allow the exchange of molecules. Basal vascular permeability describes rapid flux of small molecules such as water, salts, and gases across the endothelial barrier of capillaries. Importantly, the plasma filtrate during BVP contains very little plasma protein (McDonald et al., 1999; Nagy et al., 2008; Dvorak, 2010; Dvorak, 2021). However, during AVH or CVH, there is extensive protein extravasation with protein concentration of the exudate near plasma levels (Majno et al., 1961). In the events of AVH, the protein exudate can be beneficial in that it promotes angiogenesis and induces migration and activation of inflammatory cells like neutrophils and monocytes to clear dead tissue or pathogens. Crucially, AVH is self-limiting unlike CVH which can occur from chronic exposure to permeabilizing factors in cases of tumors or chronic inflammation (Nagy et al., 2008; Dvorak, 2010). Persistent increased protein exudate is problematic because it can result in edema and increased interstitial pressure. Accumulation of interstitial fluid is damaging to tissue health because it increases the diffusion distance for nutrients and waste products, which may compromise cellular metabolism (Scallan et al., 2010). In addition, many organs are surrounded by unyielding fascial sheaths and consequently have limited ability to expand their interstitial space. This means that relatively small increments in fluid induce large increases in interstitial pressure. Furthermore, if hyperpermeability is left to persist and edema increases, the vascular transmural pressure gradient decreases and capillaries can be physically compressed, thereby reducing tissue perfusion needed for nutrient and waste exchange. Chronically elevated extracardiac PEx after MI as demonstrated in our study highlights the clinical significance of assessing multiorgan health after MI especially upon development of CHF. The extent of extravasation of plasma solvent and solute depends on pressure and concentration gradients, hemodynamic forces, intrinsic permeability of the vascular wall (Dvorak, 2010), and inflammatory status of a given tissue (McDonald et al., 1999). By extrapolating PEx using EB concentration, we can account for the sum total of all these factors, however, we cannot be certain which mechanism is primarily responsible for the observed PEx of an organ.

Chronic heart failure can occur secondary to earlier cardiac events like MI (Kemp and Conte, 2012). The initial infarct causes early inflammatory activation which is a necessary event for the transition to later reparative stages (Guo and Dipietro, 2010; Kain et al., 2014; Prabhu et al., 2016). The reparative stage begins around 72 h in mice (Dewald et al., 2004) and is marked with significant collagen deposition (Cleutjens et al., 1995) and myofibroblast activation (Sun and Weber, 1996) that lead to the development of fibrotic tissue (Sun and Weber, 2000; Sun et al., 2002; Dewald et al., 2004) and continued scar development. These adverse changes culminate in LV remodeling and ultimately hemodynamic perturbances that include elevated cardiac filling pressures, reduced cardiac output, and central venous congestion that are characteristic of CHF (Verbrugge et al., 2020). Based on these considerations, we predicted that our MI rat model would cause inflammatory-induced AVH in the heart and lungs while hemodynamic alterations characteristic of CHF would drive CVH in all organs.

Our rat data closely correspond with murine findings in that PEx of all regions of the heart peaked at 3 days with levels diminishing thereafter, suggesting resolution of inflammation and the initiation of repair. Of note, this may be a species-specific response; dogs demonstrated a more delayed response, with the reparative stage not beginning until 7 days (Dewald et al., 2004). Of intertest, PEx is significantly reduced in the remote region at 10 weeks but reemerges at 4 months which may be linked to the development of CHF and continued remodeling of the heart.

Our data indicate that PEx of the infarcted area is not resolved 4 months after MI. We believe that the initial PEx (3 days) is a direct result of cardiac inflammation and that chronic PEx is the result of cardiac remodeling. Angiogenic factors like vascular endothelial growth factor (VEGF), which have been shown to increase VP (Nagy et al., 2008), are highly expressed in the peripheral zone of the infarct following MI. Subsequently, the peripheral zone becomes highly vascularized just 3-days post MI (Sun et al., 2002; Rog-Zielinska et al., 2016) and remains highly vascularized over 8 weeks (Kalk et al., 1997; Sun and Weber, 2000) suggesting angiogenesis. Neo-vascularization would support and contribute to the creation of dense fibrillar collagen networks that are found in the infarct scar 4 weeks after MI (Sun et al., 2002). Unlike other tissues the heart demonstrates continued collagen deposition (Cleutjens et al., 1995) and myofibroblast activation (Sun and Weber, 1996) for prolonged periods of time–up to several months in rats–that would require nutrients from newly created vascular beds. We speculate that blood flow and exudate in the peripheral zone may diffuse into the fibrotic scar. Little is known about the capacity of vessels to deliver nutrients and diffusion of such nutrients to the fibrotic scar, but our data indicate that PEx of the infarct is not resolved 4 months after MI.

Given the lungs anatomic and physiological proximity to the heart, it can be reasoned that the lungs would be the first and most severely impacted extracardiac organ following an MI. Our data corroborate with this line of thinking evidenced by significant acute pulmonary PEx. Inconsistent to the heart, the lung did not experience diminishment of PEx over time. It may even be increasing, but due to lack of statistical significance we cannot be certain.

As far as lung PEx is concerned, we believe the same general mechanisms are in play: initial inflammation followed by hemodynamic perturbance. Small infarcts that do not lead to elevated left ventricular end diastolic pressure induced neutrophil infiltration into the lung (Yan et al., 2014). This suggests that cardiac damage is sufficient to promote lung inflammation. However, as time goes on, diminished heart function and left-sided heart failure lead to pulmonary hypertension and an increase in pulmonary arterial wedge pressure (PAWP) (Kuebler et al., 1999; Konstam et al., 2018) both of which have deleterious effects on pulmonary function including increased plasma exudate and edema. Pulmonary hypertension can also progress to poorer right ventricular reserve, and when coupled with an increase in PAWP, will drastically worsen lung congestion (Reddy et al., 2019). Lymph flow may increase by a factor of 20–30 under sustained conditions of increased filtration attributable to elevated capillary pressure (Uhley et al., 1962). However, the increased levels of chronic PEx indicate pathologic VP that could overwhelm the lymphatic system. Even if high levels of edema are absent, small accumulations of interstitial fluid within the lungs will dramatically impair gas exchange and potentially interfere with other organ systems evidenced by the fact that lung injury can induce extrapulmonary organ PEx (Kitzerow et al., 2022).

The interplay of the heart and kidney cannot be understated; the kidneys provide clearance of waste products, maintenance of volume and electrolyte homeostasis, and modify neuroendocrine activities. Our data show elevated PEx in the kidney occurring at 4 months post MI. These data are consistent with the trend of renal pro-inflammatory/injury markers post MI (Xia et al., 2022). The lack of PEx at 10 weeks post MI may be due to a renal compensatory response that seeks to normalize cardiac output and arterial pressure post MI. The compensatory stages of heart failure may normalize kidney PEx, but upon CHF and development of elevated central venous pressure (CVP), PEx resumes due to elevated hydrostatic pressure in renal post-capillary venules. Unlike other abdominal organs, the kidney is intimately encased in a fibrous capsule causing additional plasma leakage and interstitial pressure particularly damaging and producing accelerated and irreversible loss of nephrons (Schroten et al., 2016) and promoting renal failure.

The current study shows that PEx of the intestines and pancreas occurs after liver PEx begins, 3 days post MI. This suggests that liver dysfunction precedes and may contribute to dysfunction of other digestive organs. Of the GI tract, only the duodenum and cecum demonstrated significant levels of PEx at 4 months and 10 weeks, respectively, while the ileum did not. Interestingly, the pancreas was the only digestive organ that presented significant worsening of PEx as time progressed and exhibited the highest levels of PEx of any organ including the heart. Each of these organs possesses fenestrated capillaries (Milici et al., 1985; Griffin and Gao, 2017; Burganova et al., 2021) so anatomical capillary structure does not explain the observed differences.

CHF is associated with chronic pancreatitis (Nikolic et al., 2019; Hiraiwa et al., 2022), congestive hepatopathy (Seeto et al., 2000; Sundaram and Fang, 2016), protein-losing enteropathy (Chan et al., 1999), malnutrition, and loss of appetite. Due to its development in the chronic stages of CHF we believe that PEx is caused, in part, by CVP elevation. The liver is especially sensitive to increased CVP (Seeto et al., 2000) due to the hepatic portal system. Elevated CVP increases the hydrostatic pressure of post-capillary venules and promotes VP and protein exudate in the liver because of their connections to the portal system, the intestines, pancreas, and spleen. Increased CVP also increases liver lymph flux which stresses the retroperitoneal lymphatic system (Itkin et al., 2020) which can rupture intestinal lacteals and further promote gut edema (Sundaram and Fang, 2016). Acute PEx may not occur in the gut due to its relatively high tolerance to reductions in blood flow and resistance to hypoxia (Seeto et al., 2000; Zheng et al., 2015) implying that these tissues most likely do not exhibit severe oxidative or pro-inflammatory responses that increase VP due to acutely diminished cardiac output. However, severe intestinal hypoperfusion and mucosal ischemia, have been shown to increase bacterial translocation and serum endotoxins (Niebauer et al., 1999; Krack et al., 2004) suggesting increased vascular permeability. Although the peritoneal cavity has high expansive abilities, unrestrained transcapillary filtration leads to exudation of interstitial fluid into the gut lumen (Granger, 1981) and, together with pancreatic exocrine deficiency often seen in CHF (Xia et al., 2017), could explain malabsorption and loss of appetite.

The spleen exhibits significant PEx starting at 10 weeks. Levels rise significantly higher at 4 months. We expected the spleen to show PEx at 3 days due to the immune interactions between the spleen and heart. There appears to be an upward trend, but unlike the other organs, the normal spleen demonstrates relatively high levels of PEx. Even looking at the spleen macroscopically, sham and MI look very similar.

Spleen activity after MI has received much attention due to the spleen’s importance in the development of leukocytes and its ability to respond to distant tissue injury (Swirski et al., 2009). In the case of MI, cardiac tissue damage has been shown to trigger the release of splenic immune cells, which contribute to cardiac remodeling and heart failure (Van Linthout and Tschope; Prabhu, 2018; Tian et al., 2016). Elevated VP increases mobilization of immune cells. Because VP and PEx are interrelated, we hypothesized that PEx would be enhanced 3 days following MI. Swirski et al. (Swirski et al., 2009), demonstrated splenic immune mobilization 1 day after MI, suggesting that VP may only be momentarily enhanced. However, splenic PEx is not only heightened at 4 months but increasing, which may portend an inflammatory response. Chronic heart failure has been shown to induce splenomegaly as measured by volume (Fujinami et al., 2018) and mass (Tonelli et al., 2013), and stimulate white pulp and marginal zone hypertrophy (Prabhu, 2018). Alternatively, the chronic production of splenic PEx may be the result of elevated CVP much like the other abdominal organs. As discussed above, portal hypertension can induce increases in hydrostatic pressure of post-capillary venules leading to PEx and edema. Due to their lymphatic connections via the periaortic nodes (Mikhael and Khan, 2022), liver lymph pathology can also contribute to spleen PEx.

### Limitations

One of the hallmarks of CHF is elevated CVP. However, CVP was not measured in our permanent MI model, so we can only speculate that elevated CVP contributed to increased levels of PEx in each organ. In addition, lymphatics play an important role in clearing extravasated materials as well as contributing to the exudate. By measuring circulatory PEx by EB we are not able to determine the extent to which lymphatic efflux contributes to overall organ extravasate. In addition, by using EB to extrapolate PEx, we cannot determine the exact mechanisms that are occurring and driving the observed PEx of an organ. Lastly, all experiments were conducted in adult male SD rats. Further studies are required to elucidate if divergences in multiorgan PEx after MI occur due to sex differences.

## Conclusion

Vascular permeability is critical for any organ function since the tight regulation of protein extravasation helps to maintain homeostasis. Persistent increased protein exudate limits the diffusion efficiency of nutrients and waste, increases interstitial pressure that can collapse capillaries and ultimately impair organ function. Increased vascular permeability could also cause organ inflammation or edema. Plasma extravasation in varying degrees is present in all organs following MI. Due to varying timepoints of PEx initiation and intervals, the mechanisms that promote extravasation, such as cardiac inflammation, hypoperfusion, or venous pressure most likely differ among organs. This is the first study to our knowledge documenting the time varying nature of PEx following MI and the development of CHF. We believe that PEx could be an independent risk factor to predict the MI-induced cardiac and extracardiac organ dysfunction.

## Data Availability

The raw data supporting the conclusion of this article will be made available by the authors, without undue reservation.
